# Nuclear deformation acts as a mechanical switch to drive breast cancer cell migration in a confined microenvironment

**DOI:** 10.7150/thno.119211

**Published:** 2026-01-01

**Authors:** Meng Wang, Xiaodie Li, Boyang Li, Hanyu Guo, Zihan Zhao, Xiyue Sun, Wei Yan, Yubo Tan, Jinyi Liu, Yungchang Chen, Shun Li, Tingting Li, Xiaozhen Dai, Xiang Qin, Yiyao Liu

**Affiliations:** 1 Department of Medical Oncology, Sichuan Clinical Research Center for Cancer, Sichuan Cancer Hospital & Institute, Sichuan Cancer Center, and School of Life Science and Technology, University of Electronic Science and Technology of China, Chengdu 610054, P R. China.; 2 Key Laboratory of Target Discovery and Protein Drug Development in Major Diseases of Sichuan Higher Education Institutes, Chengdu Medical College, Chengdu 610500, P. R. China.; 3 Department of Otorhinolaryngology, Hospital of Chengdu University of Traditional Chinese Medicine, Chengdu 610072, P. R. China.; 4 Traditional Chinese Medicine (TCM) Prevention and Treatment of Metabolic and Chronic Diseases Key Laboratory of Sichuan Province, Hospital of Chengdu University of Traditional Chinese Medicine, Chengdu 610072, Sichuan, P R. China.; 5 Department of Urology, Deyang People's Hospital, Deyang, Sichuan 618099, P. R. China.

**Keywords:** confined migration, nuclear deformation, mechanotransduction, cPLA2-AA pathway, myosin

## Abstract

**Background:** Tumour metastasis is the primary cause of high mortality in cancer patients, and the confined migration of cancer cells is the key step in successful metastasis. However, the biomechanical properties of cancer cells during confined migration and the associated mechanotransduction mechanisms remain elusive. In this study, a hydrogel-based microchannel platform was used to investigate the migratory behaviours of breast cancer cells in wide, medium, and narrow microchannels.

**Methods:** Using fluorescence microscopy, we initially characterized MDA-MB-231 breast cancer cells cultured in three distinct hydrogel-based microchannel systems and assessed both whole-cell and nuclear morphology. In parallel, cell migration dynamics were quantified via time-lapse imaging. Immunofluorescence and laser confocal imaging were subsequently employed to systematically analyse the degree of nuclear envelope unfolding and the differential expression of Piezo1. To elucidate the force-sensing mechanism, live-cell calcium imaging was performed to record responses to mechanical stimuli. Ultimately, by constructing plasmids to regulate Lamin A/C expression (knockdown or overexpression) specifically, we demonstrated its role in controlling restricted migration through targeted interference with nuclear shape changes.

**Results:** The results demonstrated that the breast cancer cells displayed the strongest motility in narrow microchannels. Moreover, upon confinement-induced nuclear deformation, the nuclear membranes unfold and tense, which acts as a mechanical switch to facilitate the rapid migration of breast cancer cells in narrow microchannels. Further investigation revealed that the mechanosensitive ion channel Piezo1 was activated on breast cancer cells in narrow microchannels, thereby accelerating calcium influx. This process not only maintained nuclear membrane tension but also activated the cytosolic calcium-dependent phospholipase A2 (cPLA2)-arachidonic acid (AA) pathway, enhancing cell migration via increased myosin II-driven contractility.

**Conclusions:** This study demonstrates the fundamental importance of nuclear deformation and mechanotransduction in cancer cell migration, providing new perspectives for the development of therapeutic approaches that target nuclear mechanics to inhibit metastatic progression.

## Introduction

Tumour metastasis represents a major therapeutic challenge and is characterized by the complexity of the metastatic process and microenvironmental heterogeneity. This complex cascade typically involves multiple steps: tumour cells detach from the primary site, breach the basement membrane, invade blood or lymphatic vessels, circulate throughout the body, and ultimately colonize distant organs 1. Understanding the underlying mechanisms of tumour metastasis and its complex interactions with the tumour microenvironment (TME) is essential for developing effective inhibition strategies. According to the cancer conceptual framework 2, the TME serves as both a dynamic scaffold for tumour cell remodelling and an active participant in driving malignant progression 3. These alterations are primarily characterized by aberrant biomechanical properties, including elevated solid stress 4, enhanced matrix stiffness 5, increased interstitial fluid pressure 6, and modified surface topography 7. Such biomechanical dysregulation not only contributes to treatment resistance but also directly modulates cell migration through mechanical confinement. Intravital microscopy further revealed that migrating cancer cells preferentially utilize pre-existing tissue pores or tunnel-like confined spaces rather than solely relying on active stromal degradation 8-11. This structural adaptability-driven migration strategy markedly enhances efficiency while maintaining stable trajectory diameters 12-14, a feature critical for successful tumour metastasis *in vivo* 15-17. These findings provide insight into the limited clinical efficacy of matrix metalloproteinase inhibitors 18, suggesting that targeting confined migration could offer a promising avenue for nonenzymatic therapeutic intervention.

*In vivo* observations of tumour cell metastasis have significantly advanced research methodologies, particularly in terms of the transition from traditional 2D culture to more sophisticated 3D culture systems. Compared with conventional 2D cultures, these 3D systems can more accurately mimic the mechanical and biochemical complexity of the TME 19, thereby serving as crucial tools for elucidating the physical cues (such as matrix stiffness and topology) that govern metastasis 20-24. For example, studies have demonstrated that adhesion-dependent tumour cells dynamically modulate their mobility patterns to adapt to a microenvironment with varying mechanical properties 25, 26. Further investigation revealed that localized matrix degradation becomes necessary when tumour cells navigate through fibre pores with cross-sectional areas smaller than approximately 7 μm² 27. However, current *in vitro* models still fail to fully recapitulate the hallmarks of the *in vivo* tumour microenvironment. Furthermore, the molecular mechanisms through which tumour cells sense physical confinement via mechanosensory pathways and subsequently transduce migratory signals remain poorly understood. Although extensive research has investigated the influence of microenvironmental factors on cell migration, the coupling mechanism between physical confinement and intracellular signalling remains to be systematically elucidated.

Recent investigations have improved our understanding of mechanosensing in confined migration by revealing the nucleus as an active mechanical regulator 28, 29, challenging its traditional characterization as merely a passive rate-limiting obstacle 30. Emerging evidence has demonstrated that under physiological confinement, diverse cell types—including normal cells, immune cells, and cancer cells 31 —actively navigate tight spaces through cell-autonomous morphological plasticity rather than passively endure compression. This dynamic behaviour suggests the presence of intrinsic mechanoresponsive mechanisms in which nuclear deformation events (such as Lamina remodelling and chromatin reorganization) potentially function as triggers for downstream signalling pathways. Nevertheless, fundamental questions remain unresolved regarding how nuclear mechanosensing interprets confined environments and interfaces with cytosolic mechanotransduction cascades to orchestrate migration decisions. This knowledge gap represents a critical frontier in understanding the mechanobiology of cell migration and tumour metastasis.

In this study, we propose a fundamental hypothesis: the active regulation of nuclear morphology serves as a crucial mechanical switch that governs breast cancer cell migration within confined environments. To definitively assess this hypothesis, we developed polydimethylsiloxane (PDMS) microconfinement platforms utilizing photolithographic patterning techniques integrated with biomimetic hydrogel networks to recapitulate the aberrant mechanopathological characteristics of the tumour stroma. Through comprehensive quantitative analysis of nuclear morphology, we sought to decipher the regulatory mechanisms by which confined microenvironments modulate breast cancer cell migration through mechanosensitive nuclear envelope signalling. This understanding illuminates novel therapeutic strategies for suppressing breast cancer cell metastasis.

## Materials and Methods

### Cell culture and reagents

Human metastatic MDA-MB-231 breast cancer cells were acquired from the American Type Culture Collection (ATCC). Lamin A/C, SUN2, and Piezo1 were knocked down using lentivirus (the cells were kindly provided by Suzhou Haixing Biosciences Co., Ltd.). All cells were maintained in Leibovitz's L-15 medium supplemented with 10% foetal bovine serum (FBS) and 1% penicillin/streptomycin (Thermo Fisher Scientific). Type I rat collagen (Shengyou) at a concentration of 5 mg/mL was used for the experiments. High-molecular-weight sodium alginate was purchased from Merck.

### Preparation and characterization of hydrogel microchannels

A polydimethylsiloxane (PDMS) stamp (DC-184; Dow Corning; crosslinking agent/monomer ratio of 1/10~1/9) was fabricated using a silicon master containing grooves (YW, MEMS. width: 3, 9 and 25 μm; depth: 25 μm; and length: 18 mm) created by photoetching. Afterwards, the hydrogel mixture (6% 0.2 M NaOH, 10% 10× PBS, 24% 37 mg/mL alginate, and 60% 5 mg/mL collagen all by volume thoroughly mixed in a centrifuge tube on ice) was micromoulded into a grooved substrate with a defined size using a PDMS stamp. To prevent the interference of alginate crosslinking with collagen gelation, we gelled the collagen before alginate crosslinking (37 °C for 45 min). After the microchannel formed, we subsequently added CaCl_2_ (56 mg/mL) to crosslink the alginate and carefully separated it from the PDMS stamp. Finally, a piece of hydrogel of the same material was placed on the top of the hydrogel microchannel and incubated to form 3D microchannels.

The microchannels were characterized by using a hydrogel mixture confused with fluorescent microbeads (Thermo Fisher, Cat. #F8827, 1:1000). The dimensions of the hydrogel microchannels were characterized by laser scanning confocal microscopy (LSM980, ZEISS, Germany) with 0.5 μm z-stacks at 488 nm. Images of the microchannels were analysed by ZEISS software (v. 3.8).

The hydrogel microchannels have a glass coverslip for support. For convenience, the object is removed before imaging. The hydrogel microchannels are mechanically fragile; therefore, we adopted the following approach to prevent damage caused by external forces during imaging: first, a glass coverslip (for easy handling with forceps) was placed at the bottom of the culture dish, and then the hydrogel microchannels were fabricated on this glass coverslip. This setup ensures structural integrity during microchannel transfer and imaging.

### Cell migration experiments

Cells (5000 cells/cm^2^) were seeded in the hydrogel grooves (30 min) in an incubator. Cells outside the grooves were gently washed away, and the contrast was covered with a hydrogel cover of the same material. The cell nuclei were stained with Hoechst (Beyotime, 1:1000) for visualization. The preparations were transferred to a live-cell incubator equipped with a controllable cellular environment and mounted on the motorized stage of the inverted microscope (Nikon Eclipse Ti2-E, Japan) before observation and time-lapse imaging of cell migration. In our work, only isolated cells without cell‒cell contact were analysed using ImageJ (USA).

### Immunofluorescence

Cells were fixed with 4% paraformaldehyde at room temperature, permeabilized with 0.1% Triton X-100, and blocked with 1% bovine serum albumin (BSA). To visualize specific cellular components, the cells were sequentially incubated with primary and secondary antibodies. The following antibodies were used: anti-Lamin A/C (Abcam, ab232730, 1:1000), anti-Piezo1 (Thermo Fisher, Cat. #PA5-106296, 1:500), anti-Lamin B1 (Abcam, ab232730, 1:1000), anti-emerin (Abcam, ab156871, 1:200), anti-nesprin1 (Thermo Fisher, Cat. #MA5-18077, 1:200), anti-SUN1 (Abcam, ab103021, 1:500), anti-SUN2 (Abcam, ab124916, 1:1000), anti-cPLA2 (Abcam, ab227246, 1:1000), anti-p-cPLA2 (Abcam, ab53105, 1:200), anti-p-MLC (Cell Signaling Technology, Cat. #95777S, 1:500), and dilactate (DAPI, Solarbio Life Sciences, Cat. #C0060, 1:10000). Primary antibodies were incubated at 4 °C for 12 h (the cells were incubated with DAPI in a humid chamber for 10 min at room temperature). Actin was visualized with a phalloidin-iFluor^®^ 555 conjugate (AAT Bioquest. Cat. #23119, 1:1000) following secondary antibody incubation. HEPES buffer (Salario, Cat. #1090) was used for both washing and antibody dilution between steps.

### Quantification of nuclear texture

To analyse the nuclear membrane morphology, contours were traced at three distinct heights: 1/4, 1/2, and 3/4 of the distance from the base to the apex of the nucleus. These contours were converted into binary images, and MATLAB was used to measure the distances between the nuclear membrane edge and the nuclear centre. The ratio of these measurements to the minimum enclosing circle (the smallest circle that completely contains all the points in a given set) was plotted to generate waveform graphs along positional coordinates, illustrating nuclear ruffling. With the use of GraphPad Prism 8, the data were graphically represented as a function of angular degrees in a clockwise direction, referred to as the amplitude profile. After data plotting, mathematical smoothing techniques were applied to reduce the noise inherent in pixel-derived signals, enhancing data accuracy and clarity. The local maximum values (peaks) in the plot were quantified. Additionally, fluorescence images of cell nuclei were processed using ImageJ to delineate the nuclear membrane contours, resulting in binary images that were imported into MATLAB for curvature analysis, producing maps that depicted the distribution of nuclear membrane curvature.

### Image analysis

All fluorescence imaging was performed using a ZEISS confocal microscope with a z-depth of 0.3 μm. Three-dimensional rendering of cell and nuclear morphology was accomplished using Imaris (v.10.0; Oxford Instrument, Andor). The DAPI fluorescence images were computationally thresholded to determine the projected nuclear cross-sectional area. The aspect ratios for both cells and nuclei were determined by calculating the ratio of the long axis (major axis) to the short axis (minor axis). All the imaging parameters remained consistent throughout the acquisition process to facilitate cross-condition comparative analyses. Quantification of p-cPLA2 in both nuclear and cytoplasmic compartments was performed utilizing ImageJ software, which was specifically applied to the maximum intensity projections derived from the DAPI and p-cPLA2 immunofluorescence images. The nuclear region of interest (ROI) was defined through the analysis of DAPI images, while the cytoplasmic compartment was identified as the residual area encompassing the cell after nuclear ROI exclusion. The average intensity values for each ROI were used to compute nuclear-to-cytoplasmic ratio indices. For F-actin quantification, the total area demarcated by phalloidin was summed and normalized to the total cell area. Fluorescence colocalization analysis was conducted using the Coloc2 plugin in Fiji.

### Intracellular Ca^2+^ imaging

The cytoplasmic calcium concentration in living cells was monitored using the fluorescent dye Cal-520® AM (ATT Bioquest, Cat. #21130). Following the seeding of cells in hydrogel microchannels, the microchannels were incubated with a 5 μM working solution of Cal-520^®^ AM at 37 °C for 30 min. Subsequently, the cells were rinsed with Hanks' balanced salt solution (Thermo Fisher, Cat. #14025092). Fluorescence images were acquired using an LSM980 to visualize the cells, and the calcium fluorescence intensity within the cytoplasmic region of interest (ROI) was analysed using ImageJ software.

### Drug treatments

The following pharmacological agents were utilized in the experiments: 10 µM extracellular Ca^2+^ chelator BAPTA (MedChemExpress, Cat. #HY-100168), 10 µM ionomycin (IOM) directly facilitating the transport of Ca^2+^ across the plasma membrane (MedChemExpress, Cat. #HY-13434), 10 µM 2-aminoethyl diphenylborinate (2-APB) blocking stretch-activated inositol triphosphate receptors (InsP3Rs) on the endoplasmic reticulum (ER)/nuclear membranes (MedChemExpress, Cat. #HY-W009724), 20 µM Yoda1 (MedChemExpress, Cat. # HY-18723) activating the mechanosensitive ion channel protein Piezo1, 2.5 µM peptide GsMTx4 from the tarantula venom affecting mechanosensitive ion channels on the plasma membrane (MedChemExpress, Cat. #HY-P1410), and 1 µM pyrrophenone (Cayman, Cat. #13294) inhibiting the nuclear envelope stretch-sensitive enzyme cPLA2. The culture medium was supplemented with 1% DMSO (v/v) (Sigma, Cat. #D8371) in the control experiments.

### Transient transfection

Plasmids were propagated in *E. coli*, which were subsequently transformed and cultured on solid media supplemented with 1.5% to 2.0% (weight/volume) agar. Single colonies were isolated and grown, and plasmids were extracted following the protocol provided with the plasmid extraction kit (Omega Bio-Tek, Cat. #D6942. The DNA concentration was quantified spectrophotometrically, ensuring an A260/A280 ratio between 1.8 and 2.0. Cells were transfected with plasmid DNA using Lipofectamine LTX reagent (Thermo Fisher, Cat. #15338100) either transiently or stably, according to the manufacturer's protocol. Fluorescence microscopy was employed to observe the successful transfection of the plasmid with a fluorescent tag, which was then utilized for subsequent experimental investigations.

### Arachidonic acid (AA) assay

Intracellular AA levels were quantified using commercial ELISA kits (Cloud-Clone Corp, Cat. #USEB965Hu, 96T). After the expansion of the microchannels to increase the cell seeding density, equivalent cell quantities from different channels were collected. Following the manufacturer's protocol, the samples were incubated/washed on precoated plates, and the absorbance was measured at 450 nm using a microplate reader. The concentrations calculated from triplicate biological replicates were used for statistical analysis.

### Statistical analysis

GraphPad Prism 8.0 was used for all the statistical analyses. The data are presented as the mean ± standard error of the mean (SEM) or standard deviation (SD). The sample size (n) and p values are specified in the main text or figure legends. All the quantitative data subjected to statistical analysis were derived from a minimum of three independent experiments.

## Results

### Breast cancer cells exhibited the highest motility in the Nar channel

**Figure 1:**
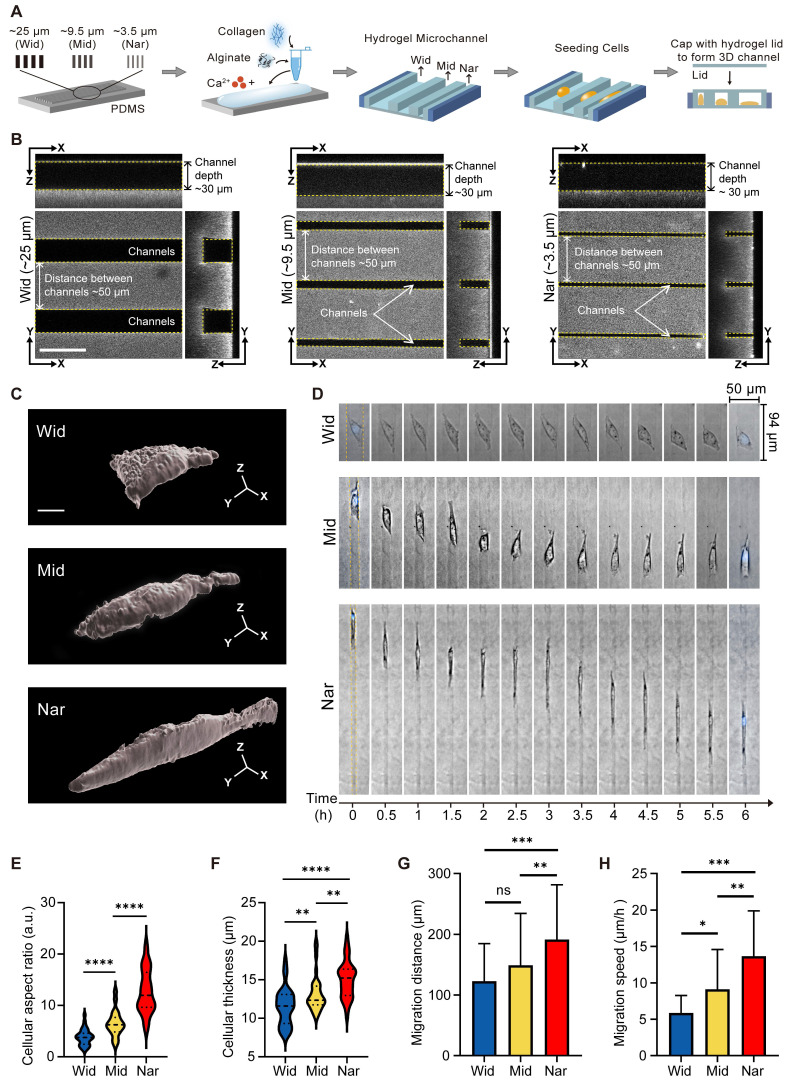
Effects of hydrogel microchannels on breast cancer cells motility. (**A**) Schematic diagram of hydrogel microchannel fabrication. (**B**) Three-view diagrams of Wid, Mid, and Nar channels. The channel edges are marked by yellow dotted lines. scale bar: 50 μm. (**C**) Three-dimensional modeling images of breast cancer cells in channels, scale bar: 5 μm. (**D**) Time-lapse image segments of breast cancer cells migration in Wid, Mid, and Nar channels. The channel edges are marked by yellow dotted lines, and nuclei stained with Hoechst (DNA, blue). (**E-H**) Statistics of breast cancer cells aspect ratio (**E**), cell thickness (**F**), migration distance (**G**), and migration speed (**H**) in Wid, Mid, and Nar channels (n ≥ 6 cells per condition), (Statistical significance: ****p < 0.0001, ***p < 0.001, **p < 0.01 and *p < 0.05, ns: not significant).

To investigate the effect of confined space on the behaviour of breast cancer cells, we constructed an *in vitro* culture model using a hydrogel-based microchannel. The width of the microchannel in this model can be regulated (see “Methods”) and was used to mimic spatial restrictions in the tumour microenvironment (Figure 1A). We chose three different widths of microchannels for the following experiments: the wide microchannel (~25 μm, termed the “Wid” channel), the midsized microchannel (~9.5 μm, termed the “Mid” channel), and the narrowest microchannel (~3.5 μm, termed the “Nar” channel; Figure 1B). Following the seeding of MDA-MB-231 cells into three distinct channels, marked variation in the morphology of the breast cancer cells was observed between the Nar and Wid channels (Figure 1C). The cells in the Nar channel demonstrated a more elongated morphology and increased cell thickness (Figure 1E-F). In addition, cells in the Nar channel exhibited the most pronounced migratory capacity, both in terms of migration distance and speed (Figure 1D, Figure 1G-H). These results indicated that individual cells in the channels could sense environmental variations and exhibited enhanced motility in a specific confined space.

### Confinement-induced nuclear deformation caused nuclear membrane unfolding

**Figure 2:**
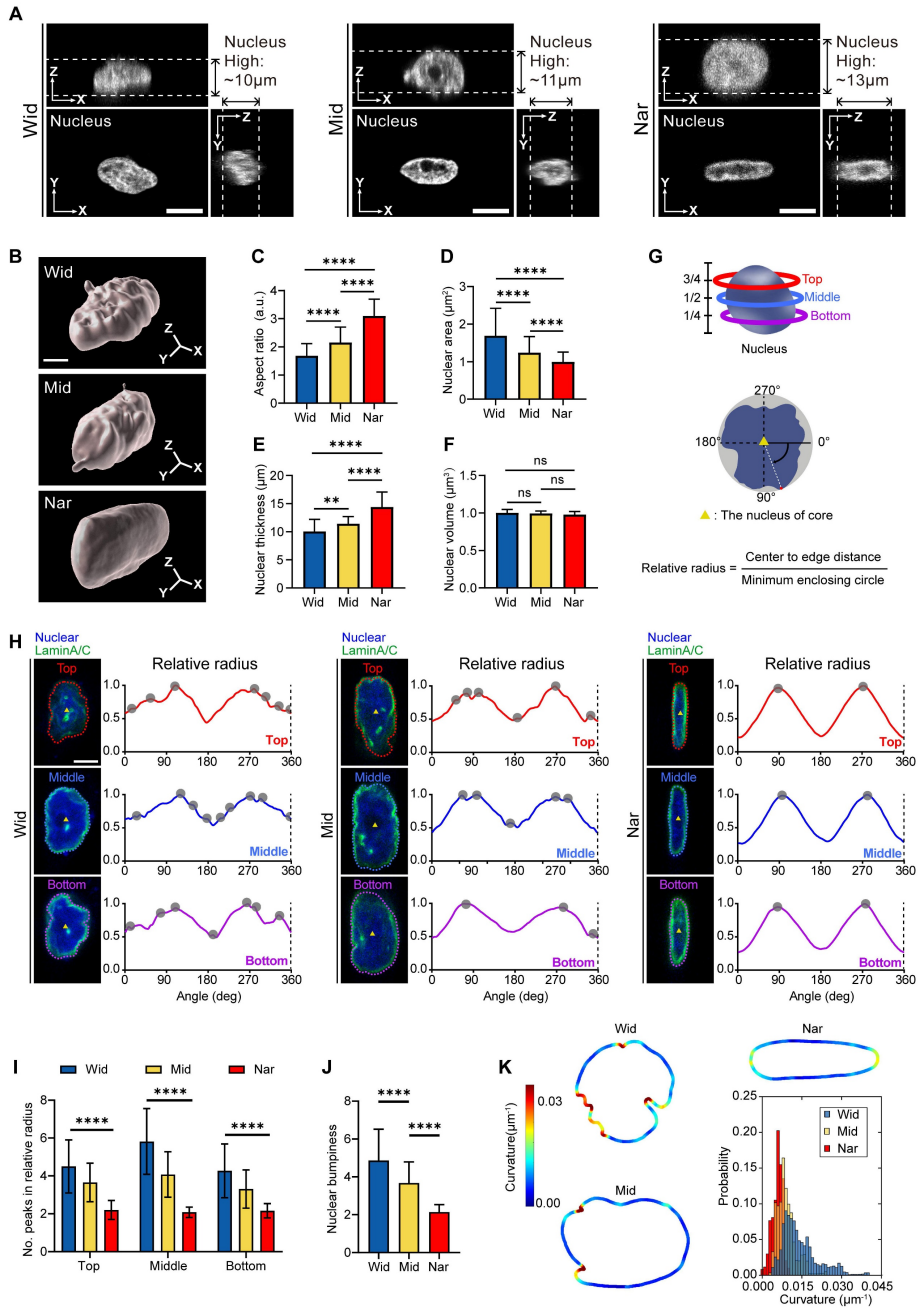
Morphological changes of MDA-MB-231 nuclei in Wid, Mid, and Nar channels. (**A**) Three-view diagrams of DAPI-stained nuclei in Wid, Mid, and Nar channels obtained through laser confocal z-stack scanning, with white dotted lines indicating nuclear height, scale bar: 10 μm. (**B**) Three-dimensional modeling images of breast cancer cells nuclei in channels, scale bar: 5 μm. (**C-F**) Statistics of nuclear aspect ratio (**C**), maximum projection area (**D**), thickness (**E**), and volume (**F**) in different channels (n ≥ 20 cells per condition). (**G**) Schematic diagram of nuclear membrane wrinkle analysis (see method). (**H**) Nuclear membrane wrinkle waveforms, tracking surface contours at three different heights show that nuclei in Wid channels have significantly more peak positions (gray dots) than those in Nar channels, indicating more irregular surfaces and wrinkles in Wid channel nuclei. Relative radius is the ratio of nuclear center-to-edge distance to the minimum enclosing circle, with upper (red), middle (blue), and lower (purple) corresponding to 75%, 50%, and 25% positions along the nuclear z-axis (**G**), scale bar: 5 μm. (**I**) Statistical analysis of relative radius peaks at each height (n ≥ 22 cells per condition). (**J**) Average peak numbers of relative radii at three different heights, termed nuclear lateral roughness (n ≥ 22 cells per condition). (**K**) Analysis of curvature value distribution for maximum nuclear cross-sectional area, with curvature value distribution chart in lower right (n ≥ 16 cells per condition), (Statistical significance: ****p < 0.0001 and **p < 0.01, ns: not significant).

The nucleus, the largest and stiffest organelle in tumour cells, has been traditionally viewed as a barrier to cell migration *in vivo* 32. However, recent studies have revealed that the nuclei are attractive for mechanotransduction owing to their unique functions, such as sensing mechanical cues 33. To determine whether the nucleus participated in cell sensing of environmental changes, we characterized the nuclear structures in different channels (Figure 2A-B). Compared with the Wid channel, the nuclear morphology projected to the XY plane showed a reduced nuclear area and an elongated nuclear shape in the Nar channel (Figure 2C-D). Although the nuclear volume remained unchanged, we found that the nuclear thickness significantly increased in the Nar channel (Figure 2E-F). Moreover, the nuclei in the Nar channel exhibited smoother surface topography, whereas those in the Wid channel displayed enhanced surface roughness with pronounced membrane irregularities (Figure 2B). To assess the extent of nuclear deformation, we examined the distribution of Lamin A/C, a key component of the nuclear lamina that is essential for nuclear mechanical stability and morphology maintenance 34. Comparing the breast cancer cell nuclei, in the Nar channel, the breast cancer cell nuclei presented only two peaks in terms of the relative radius, with each peak corresponding to the maximum circular radius along the nuclear surface (Figure 2H), whereas the cells in the Wid channel presented more than two peaks at different altitudes, indicating an irregular nuclear morphology. Further analysis of the peak numbers of breast cancer cells at different levels and average peak numbers of relative radii within three vertical strata (bottom, middle, and top) revealed the pronounced morphological irregularities specifically localized to the nuclear periphery in Wid channel nuclei (Figure 2I-J). Nuclear membrane curvature analysis also confirmed that the nuclear membrane curvature of breast cancer cells in the Wid channel was significantly greater than that of cells in both the Nar channel and the Mid channel (Figure 2K). These results demonstrate that by adopting distinct nuclear morphologies, breast cancer cells adapt to different confined spaces.

### Piezo1 in breast cancer cells was activated in the Nar channel

**Figure 3:**
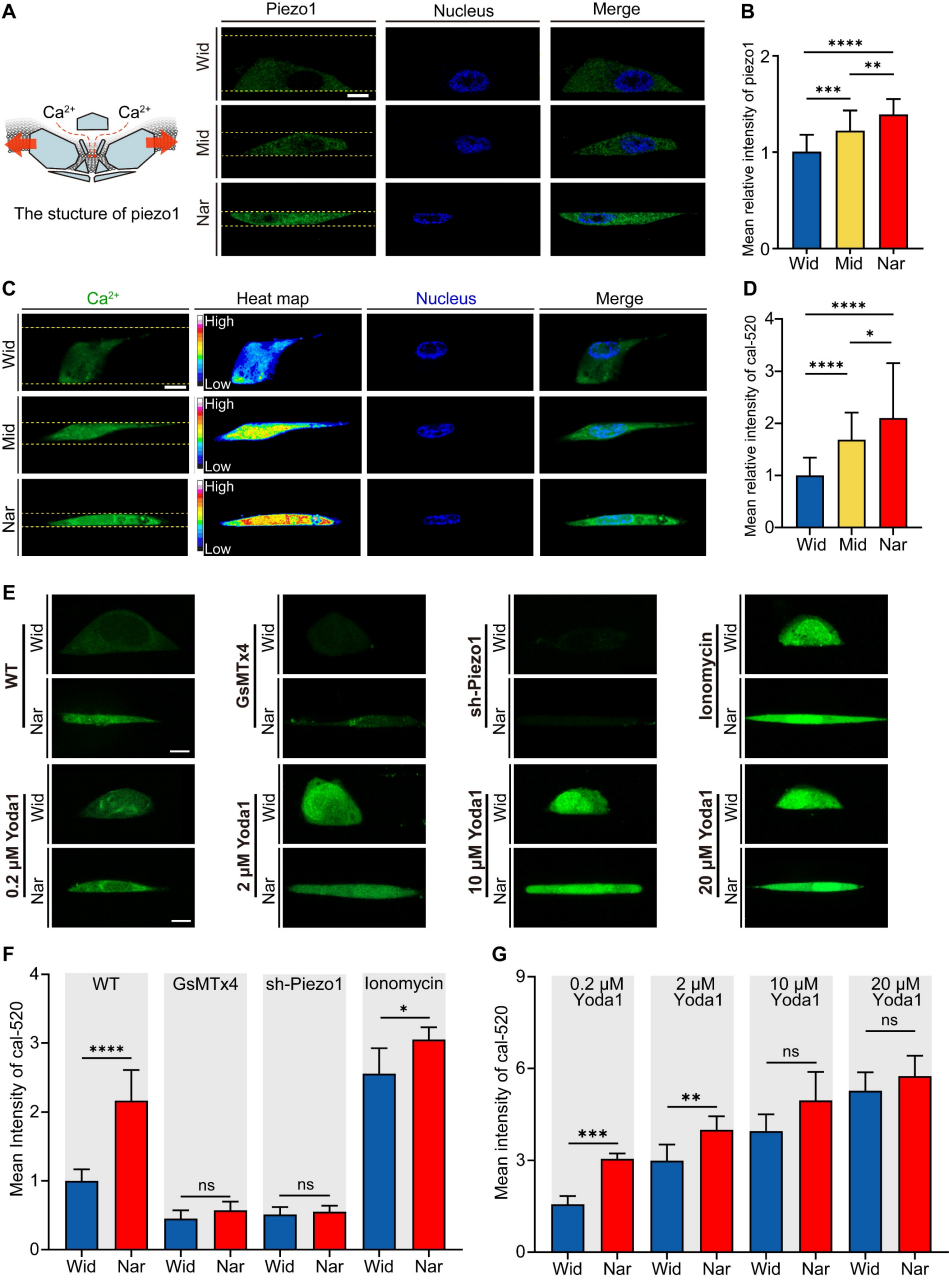
Impact of Spatial Confinement on the Mechanosensitive Ion Channel Protein Piezo1. (**A**) Left: schematic representation of Piezo1 structure; Right: Representative fluorescence micrographs of Piezo1 expression in cells confined within Wide, Mid, and Nar channels. DAPI (blue) shows nuclear staining, Piezo1 (green), and channel boundaries are indicated by yellow dotted lines. Scale bar: 10 μm. (**B**) Quantitative analysis of average relative Piezo1 fluorescence intensity across Wid, Mid, and Nar channels (n ≥ 45 cells per condition). (**C**) Representative live-cell calcium imaging showing intracellular Ca^2+^ levels. Channel boundaries are marked by yellow dotted lines. Scale bar: 10 μm. (**D**) Quantitative analysis of relative intracellular Ca^2+^ fluorescence intensity in Wid, Mid, and Nar channels (n ≥ 43 cells per condition). (**E**) Representative live-cell calcium imaging showing intracellular Ca^2+^ levels in Wid and Nar channels following Piezo1 modulation through pharmacological intervention or genetic manipulation. Scale bar: 10 μm. (**F-G**) Quantitative analysis of relative intracellular Ca^2+^ fluorescence intensity following drug treatment and plasmid transfection (n ≥ 15 cells per condition). (Statistical significance: ****p < 0.0001, ***p < 0.001, **p < 0.01 and *p < 0.05, ns: not significant).

To investigate how breast cancer cells respond to mechanical cues, we analysed the expression levels of Piezo1, a mechanosensitive ion channel protein crucial for sensing environmental changes 35. We evaluated Piezo1 activation states under different confinement conditions by measuring intracellular Ca^2+^ concentrations. Our results revealed that cells in the Nar channel exhibited significantly higher Piezo1 expression levels (Figure 3A-B) and higher intracellular calcium concentrations (Figure 3C-D), as demonstrated by live-cell calcium imaging. These findings indicate enhanced functional activity of Piezo1 in cells within the Nar channel. To further examine Piezo1 sensitivity in confined channels, we employed GsMTx4, a specific mechanosensitive ion channel inhibitor. GsMTx4 treatment effectively blocked Ca²⁺ influx in both the Wid and Nar channels (Figure 3E). Consistent results were obtained in Piezo1 knockdown cells (sh-Piezo1). When stimulated with ionomycin, cells in both the Wid and Nar channels showed significant increases in Ca²⁺ levels. Additionally, cells were treated with Yoda1, a selective chemical agonist of Piezo1. Similar effects were observed (Figure 3F-G). Collectively, these findings demonstrated that Piezo1 activation in the Nar channel transforms mechanical signals into biochemical signals through elevated intracellular Ca^2+^ concentrations.

### Increased extracellular Ca²⁺ influx maintained nuclear membrane expansion in the Nar channel

**Figure 4:**
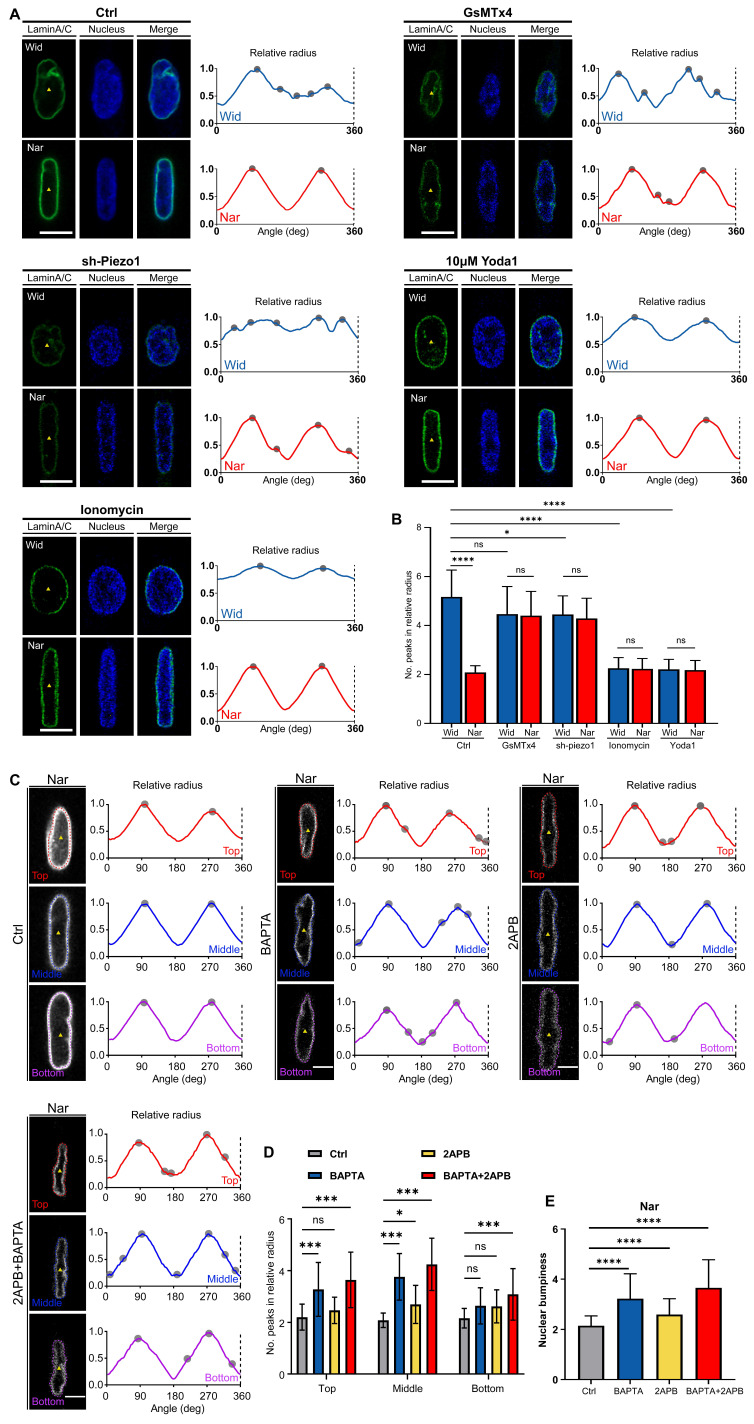
Breast cancer cells primarily modulate nuclear morphology through Piezo1-mediated extracellular Ca²⁺ influx in Nar channel. (**A**) Fluorescence images of nucleus (blue) and nuclear membrane protein Lamin A/C (green) following Piezo1 with Piezo1 inhibition or activation in Wid and Nar channels. Yellow triangles indicate nuclear cross-section centroids. Right: nuclear membrane wrinkle waveforms, and tracking surface contours show that nuclei with inhibition of Piezo1 have significantly more peak positions (gray dots) than those that increase Piezo1 activity. Scale bar: 10 μm. (**B**) Statistical analysis of relative nuclear radius peak values following Piezo1 inhibition or activation in Wid and Nar channels. (**C**) Immunofluorescence staining of Lamin A/C after chelating endoplasmic reticulum or extracellular Ca²⁺, revealing nuclear membrane morphology at different heights in confined channels. Yellow triangles indicate nuclear cross-section centroids. Right: nuclear membrane wrinkle waveforms. Scale bar: 5 μm. (**D**) Statistical analysis of relative radius peak values at each nuclear height. (**E**) Average peak values of relative radius at three different heights, quantified as nuclear lateral roughness (n ≥ 15 cells per condition), (Statistical significance: ****p < 0.0001, ***p < 0.001 and *p < 0.05, ns: not significant).

Although Piezo1 activity is enhanced in confined spaces, leading to elevated intracellular Ca²⁺ levels, the relationship between this elevation and subsequent changes in nuclear morphology remains unclear. To determine whether increased Ca²⁺ influx affects nuclear deformation, we systematically assessed nuclear periphery irregularity in the Wid and Nar channels. We observed an increase in nuclear membrane folds following Piezo1 knockdown (sh-Piezo1) or GsMTx4 treatment, both of which inhibited intracellular Ca²⁺ influx. Conversely, the nuclear membrane unfolded when treated with ionomycin or Yoda1, regardless of channel type, indicating its responsiveness to mechanochemical signals (Figure 4A and C).

To elucidate the calcium sources that drive confinement-induced nuclear membrane smoothing, we inhibited extracellular calcium influx (BAPTA) or endoplasmic reticulum calcium release (2-APB). Quantitative analysis revealed that extracellular calcium chelation exacerbated nuclear membrane folding with increased perimeter irregularity (Figure 4B, Figure 4D-E). These observations aligned with the aforementioned findings and further confirmed the critical role of Piezo1 in the Nar channel in mechanical signal transduction. In conclusion, our data revealed that breast cancer cells dynamically modulated Piezo1 activity in response to mechanical cues, with Piezo1-mediated extracellular Ca^2+^ influx emerging as a critical regulator of nuclear morphological adaptations.

### Ca²⁺-dependent regulation of nuclear morphology requires Lamin A/C and SUN2

**Figure 5:**
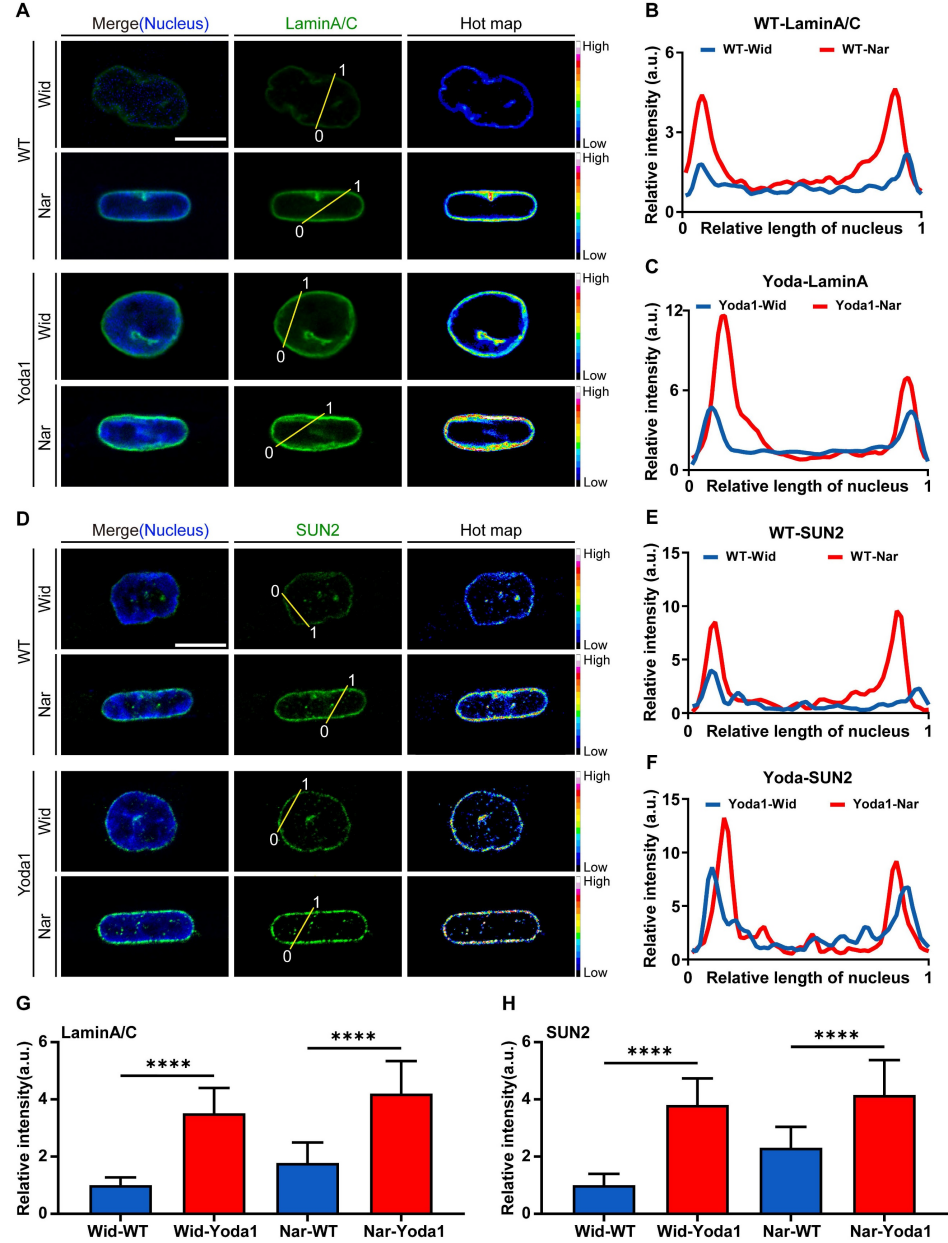
Expression of Lamin A/C or SUN2 was regulated by cytosolic Ca^2+^ concentration. (**A-C**) Fluorescence images of nuclear membrane protein Lamin A/C (green) and nucleus (blue) in Wid and Nar channels with or without Yoda1 treatment. Scale bar: 10 μm (**A**)**.** Normalized fluorescence intensity analysis along yellow lines in (**A**), showing relative fluorescence intensity at relative nuclear lengths without (**B**) or with (**B**) Yoda1 treatment. (**D-F**) Fluorescence images of nuclear membrane protein SUN2 (green) and nucleus (blue) in Wid and Nar channels with or without Yoda1 treatment. Scale bar: 10 μm (**D**)**.** Normalized fluorescence intensity analysis along yellow lines in (**D**), showing relative fluorescence intensity at relative nuclear lengths without (**E**) or with (**F**) Yoda1 treatment. (**G**) Statistical analysis of relative Lamin A/C fluorescence intensity at nuclear membrane in Wid and Nar channels with or without Yoda1 treatment (n ≥ 22 cells per condition). (**H**) Statistical analysis of relative SUN2 fluorescence intensity at nuclear membrane in Wid and Nar channels with or without Yoda1 treatment (n ≥ 46 cells per condition), (Statistical significance: ****p < 0.0001).

Nuclear morphology is determined by the mechanical properties of nuclear envelopes, particularly nuclear membrane proteins 36. The linker of the nucleoskeleton and cytoskeleton (LINC) complex plays a pivotal role in cytoplasm-to-nucleus signal transduction, serving as a bridge between the cytoskeleton and the nuclear skeleton 37. To directly validate whether Ca²⁺ affects nuclear morphology by regulating nuclear envelope proteins or components of the LINC complex, we screened and compared the expression levels of several principal components of nuclear membrane proteins and the LINC complex following Yoda1 treatment under 2D culture conditions. We observed substantial upregulation of Lamin A/C, SUN2, and Nesprin1; conversely, Lamin B1, SUN1, and Nesprin2 exhibited minimal changes in response to elevated Ca²⁺ levels (Figure. S1 and S2). Similar patterns were observed in both the Wid and Nar channels (Figure 5A-F). Furthermore, the expression levels of lamin A/C and SUN2 were significantly greater in the Nar channel than in the Wid channel (Figure 5G-H). Collectively, these results demonstrated that breast cancer cells respond to changes in cytosolic Ca²⁺ concentrations by modulating the expression of lamin A/C and SUN2, thereby sensing and responding to mechanical signals.

### The cPLA2-AA pathway was activated by confinement-induced nuclear deformation and Ca²⁺ influx, promoting actomyosin contractility

**Figure 6:**
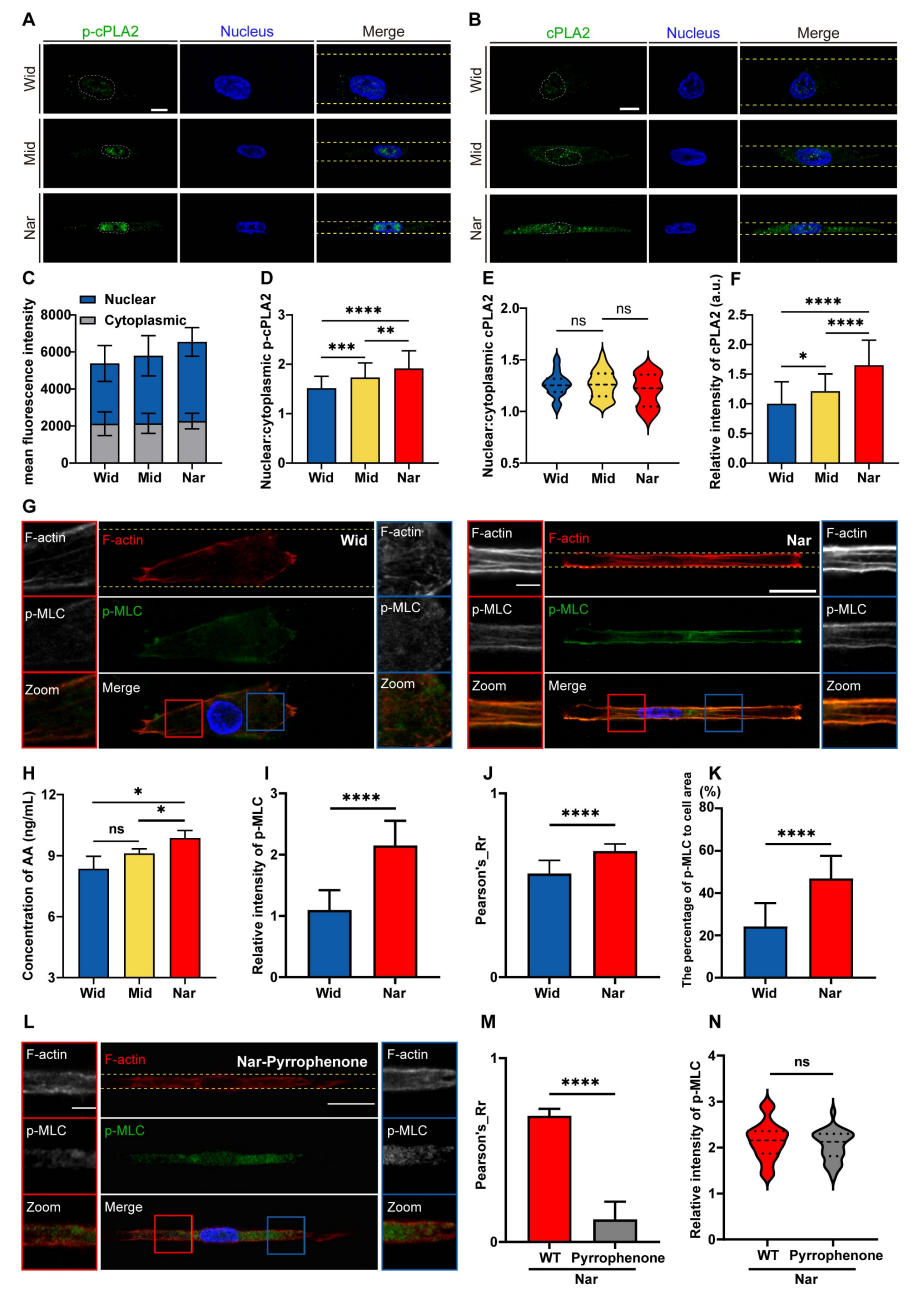
Effects of confined space on the cPLA2-AA pathway in breast cancer cells. (**A**) Fluorescence images showing p-cPLA2 (green) and nucleus (blue) staining in Wid, Mid, and Nar channels. The nuclear boundaries are marked by white dotted lines and the channel edges are marked by yellow dotted lines, scale bar: 10 μm. (**B**) Fluorescence images showing cPLA2 (green) and nucleus (blue) staining in Wid, Mid, and Nar channels. The nuclear boundaries are marked by white dotted lines and the channel edges are marked by yellow dotted lines, scale bar: 10 μm. (**C**) Statistical analysis of p-cPLA2 mean fluorescence intensity in nucleus versus cytoplasm (n ≥ 35 cells per condition). (**D**) Analysis of nuclear-to-cytoplasmic ratio of p-cPLA2 fluorescence intensity (n ≥ 35 cells per condition). (**E**) Analysis of nuclear-to-cytoplasmic ratio of cPLA2 fluorescence intensity (n ≥ 23 cells per condition). (**F**) Statistical analysis of relative cPLA2 fluorescence intensity in Wid, Mid, and Nar channels (n ≥ 23 cells per condition). (**G**) Fluorescence images of F-actin (red), p-MLC (green), and nucleus (blue) staining in Wid and Nar channels. The channel edges are marked by yellow dotted lines, scale bar: 20 μm; magnified images of corresponding colored boxes shown, scale bar: 5 μm. (**H**) Normalized statistical analysis of AA production in Wid, Mid, and Nar channels using detection kit. (**I**) Statistical analysis of relative p-MLC fluorescence intensity in Wid and Nar channels (n ≥ 37 cells per condition). (**J**) Statistical analysis of F-actin and p-MLC fluorescence co-localization correlation (n ≥ 37 cells per condition). (**K**) Statistical analysis of p-MLC fluorescence distribution area relative to cell cross-sectional area (n ≥ 37 cells per condition). (**L**) Fluorescence imaging of F-actin and p-MLC in Nar channel, cells were treated with cPLA2 inhibitor Pyrrophenone, the channel edges are marked by yellow dotted lines, scale bar: 20 μm; magnified images of corresponding colored boxes shown, scale bar is 5 μm. (**M**) Statistical analysis of F-actin and p-MLC co-localization correlation in Nar channel, cells were treated with cPLA2 inhibitor Pyrrophenone (n ≥ 30 cells per condition). (**N**) Statistical analysis of relative p-MLC fluorescence intensity in Nar channel, cells were treated with cPLA2 inhibitor Pyrrophenone (n ≥ 30 cells per condition), (Statistical significance: ****p < 0.0001, ***p < 0.001, **p < 0.01 and *p < 0.05, ns: not significant).

After preliminarily elucidating the signalling process from mechanical signal detection to nuclear remodelling, we sought to decipher how these mechanochemical signals connect to the functional output of confined migration. Given the critical role of the cPLA2-AA pathway in orchestrating membrane dynamics and contractile forces, we hypothesized that this lipid signalling hub serves as a critical mechanoeffector in confined migration. To determine the activation status of the cPLA2-AA pathway, we quantified and spatially analysed its key components. Quantitative fluorescence imaging revealed a pronounced increase in the nuclear localization of phosphorylated cPLA2 (p-cPLA2) in the Nar channel, which was positively correlated with cPLA2 activity (Figure 6A). Although the nuclear-to-cytoplasmic ratio of total cPLA2 remained comparable between the Wid and Nar channels (Figure 6B-F), cPLA2 expression levels were significantly elevated in the Nar channel, potentially facilitating enhanced cPLA2 activity or nuclear localization of p-cPLA2. AA in cells was measured using a commercial ELISA kit, followed by spectrophotometric measurement using a microplate reader after sample incubation. Notably, the AA assay revealed heightened AA production in the Nar channel, which directly reflected the catalytic generation of this primary lipid mediator by cPLA2 (Figure 6H). The increased production of AA in the Nar channel suggested its potential involvement in regulating actomyosin contractility. To investigate this connection, we examined the phosphorylation level of the myosin light chain (p-MLC), a key effector molecule for cell contractility. Quantitative fluorescence analysis revealed significantly higher intracellular levels and a broader distribution of p-MLC in the Nar channel (Figure 6G and 6I-K). The inhibition of cPLA2 with pyrrophenone robustly suppressed the colocalization of cPLA2 with F-actin (Figure 6L-N). These observations establish that the cPLA2-AA pathway is a central regulator of myosin activity and migration, particularly in confined spaces.

**Figure 7:**
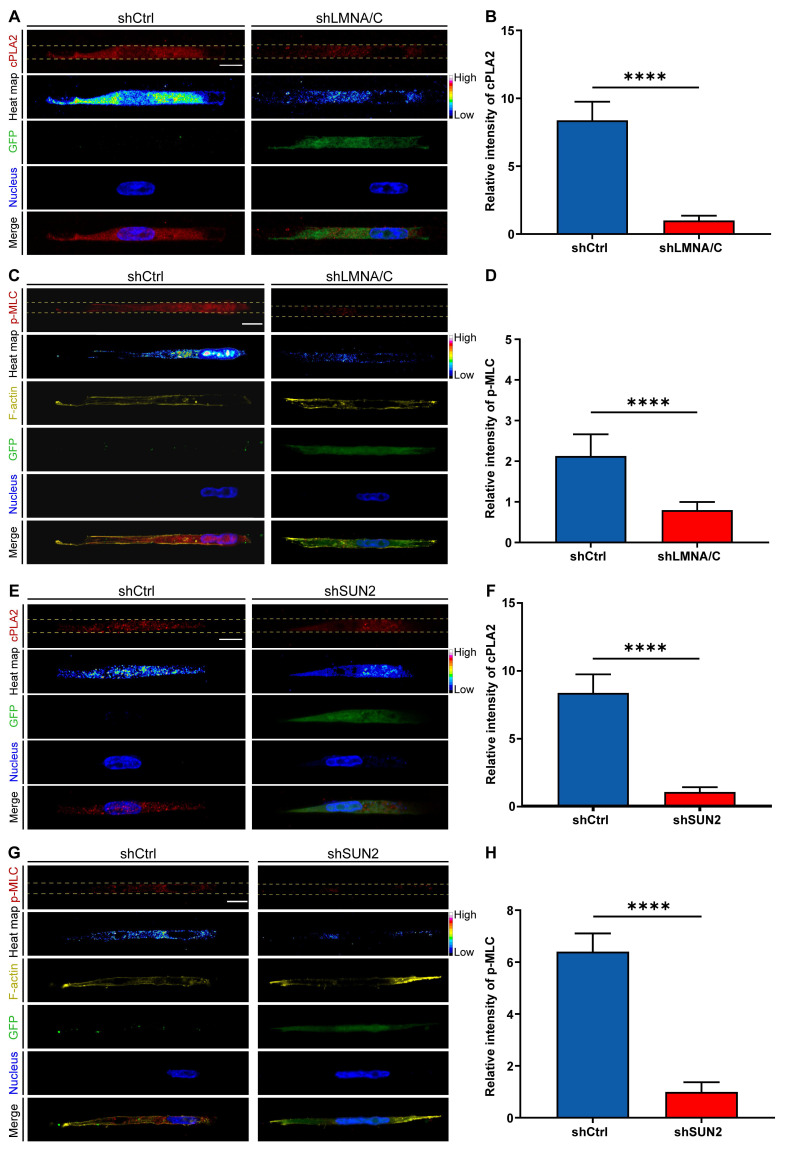
Effects of LMNA/C or SUN2 knockdown on cellular cPLA2 and p-MLC. (**A**) Fluorescence images showing cPLA2 (red) and nucleus (blue) staining in shCtrl and shLMNA/C cells after plasmid transfection in Nar channel, with GFP indicating plasmid fluorescence marker. The channel edges are marked by yellow dotted lines. (**B**) Statistical analysis of relative cPLA2 fluorescence intensity in shCtrl and shLMNA/C cells (n ≥ 5 cells per condition). (**C**) Fluorescence images showing p-MLC (red), F-actin (yellow), and nucleus (blue) staining in shCtrl and shLMNA/C cells after plasmid transfection in Nar channel, with GFP indicating plasmid fluorescence marker and yellow dotted lines indicating channel edges. (**D**) Statistical analysis of relative p-MLC fluorescence intensity in shCtrl and shLMNA/C cells (n ≥ 5 cells per condition). (**E**) Fluorescence images showing cPLA2 (red) and nucleus (blue) staining in shCtrl and shSUN2 cells after plasmid transfection in Nar channel, with GFP indicating plasmid fluorescence marker and yellow dotted lines indicating channel edges. (**F**) Statistical analysis of relative cPLA2 fluorescence intensity in shCtrl and shSUN2 cells (n ≥ 5 cells per condition). (**G**) Fluorescence images showing p-MLC (red), F-actin (yellow), and nucleus (blue) staining in shCtrl and shSUN2 cells after plasmid transfection in Nar channel, with GFP indicating plasmid fluorescence marker and yellow dotted lines indicating channel edges. (**H**) Statistical analysis of relative p-MLC fluorescence intensity in shCtrl and shSUN2 cells (n ≥ 5 cells per condition). All scale bar is 20 μm. (Statistical significance: ****p < 0.0001).

To further validate the role of nuclear deformation in cPLA2-AA pathway activation, we independently knocked down either Lamin A/C or SUN2 to prevent nuclear deformation and then measured cPLA2 levels and p-MLC expression. lamin A/C knockdown (shLMNA/C) significantly downregulated cPLA2 protein expression and inhibited MLC phosphorylation compared with those in the control group (shCtrl) in the Nar channel (Figure 7A-D). Similarly, SUN2 knockdown (shSUN2) had consistent effects on the Nar channel (Figure 7E-H). We subsequently investigated whether Lamin A/C overexpression (LMNA/C Overexpression) in wide channels affects the cPLA2 signalling pathway and p-MLC expression. The immunofluorescence results demonstrated that overexpression of Lamin A/C (LMNA/C Overexpression) led to a marked increase in both cPLA2 and p-MLC expression levels in breast cancer cells (Figure S3-4), suggesting nuclear-mediated regulation of cPLA2 and p-MLC expression. To investigate the potential impact of the cell nucleus on cell migration behaviour, we employed a time-lapse live cell imaging system to record the migration of live cells. Our observations revealed that Lamin A/C overexpression (LMNA Overexpression) enhanced cellular motility in the Wid channel (Figure S5), whereas Lamin A/C knockdown (shLMNA) reduced migration efficiency in the Nar channel (Figure S6). These findings indicate that inhibiting nuclear deformation suppresses the cPLA2 pathway, thereby reducing MLC phosphorylation, which diminishes cell contractility and ultimately impairs cell motility. Collectively, these data demonstrate that nuclear deformation acts as a mechanical switch in confined spaces: by triggering cPLA2 activation and subsequent AA release, nuclear deformation directly upregulates actomyosin contractility to drive cell migration. This mechanochemical coupling not only reveals how cells decode physical confinement but also provides a mechanistic paradigm for how mechanical signals are transduced into cytoskeletal plasticity.

## Discussion

**Figure 8:**
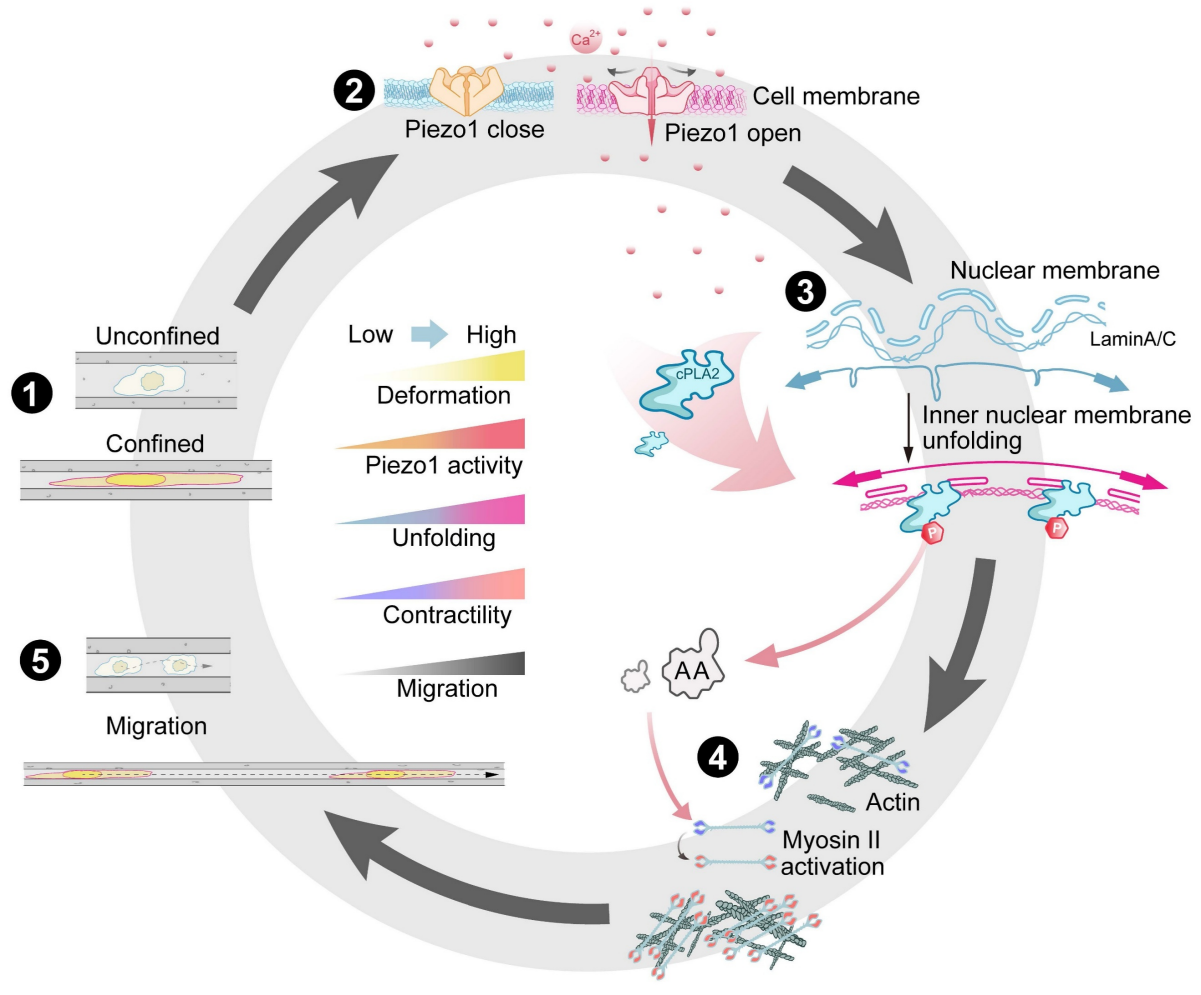
Summary of confined space effects breast cancer cell membrane structure and cell migration. (**1**) Breast cancer cells exhibit different morphological changes and differentiated motility in various confined microenvironments. (**2**) In extremely confined narrow microenvironments, the opening of mechanosensitive ion channel protein Piezo1 on the cell membrane increases, promoting intracellular calcium ion concentration elevation. (**3**) Through Lamin A/C and LINC complexes, this promotes nuclear membrane stretching, increases nuclear membrane tension, releases binding sites, favoring cPLA2 activation and nuclear localization, promoting AA production. (**4**) This activates more Myosin II, increasing intracellular contractile force. (**5**) Thus promoting cell migration in confined microenvironments, further advancing tumor malignant progression.

Biomechanical signals (e.g., matrix stiffness, shear stress, and compressive stress) dynamically regulate cellular behaviour, but the mechanisms coupling mechanosensing to signal transduction remain insufficiently illuminated. Our study on MDA-MB-231 human breast cancer cells revealed that a spatially restricted environment significantly enhances their migratory ability, highlighting their ability to dynamically adapt behaviour in response to microenvironmental constraints. The nucleus serves as a critical mechanosensory regulator 38-42, where confinement-induced deformation directly modulates chromatin organization, cellular differentiation, and migratory behaviour. We demonstrated that intracellular Ca²⁺ levels positively correlate with nuclear deformation, which is mediated through Lamin A/C and SUN2 (a LINC complex component). Mechanistically, spatial confinement activates the Piezo1 channel by increasing membrane tension, inducing calcium influx, and triggering downstream signalling (Figure 8). From a biomechanical perspective, our work delineates a fully integrated mechano-chemo-transduction network, establishing a novel framework for understanding cellular mechanosensing.

In response to the confined space, the nuclei of MDA-MB-231 cells undergo deformation with increased nuclear thickness, resulting in the stretching of the nuclear envelope. This process requires high expression of lamin A/C and SUN2 and involves Ca^2+^ signalling. We observed a robust correlation between the extent of nuclear deformation and severity of spatial confinement, which is consistent with findings in various cell lines and zebrafish embryos 28, 29, suggesting a conserved mechanosensing mechanism across species. Nuclear deformation functions as a mechanical switch, exposing binding sites that activate downstream signalling—particularly the cPLA2-AA pathway—which drives myosin contraction and optimizes cellular motility and adhesion adaptation in confined microenvironments (Figure 8). This mechanism explains how breast cancer cells maintain high migratory capacity in confined spaces, independent of classical focal adhesion/cytoskeletal sensing mechanisms. Notably, the disruption of Lamin A/C or SUN2 expression abolished nuclear deformation and subsequent dominance of myosin contraction, emphasizing the central role of nuclear mechanosensing in the single-cell model system. Interestingly, Lamin A/C depletion in epithelial tissues did not affect the nuclear morphology 43, suggesting fundamentally divergent mechanosensing mechanisms between single-cell and multicellular systems.

cPLA2, a key enzyme involved in diverse biological processes 44, plays critical roles in both physiological and pathological contexts. Notably, our results revealed elevated cPLA2 expression in the Nar channel, along with enhanced nuclear localization of phosphorylated cPLA2. Previous studies have demonstrated that cPLA2 expression is regulated by multiple factors 45, 46, including calcium ions, which primarily regulate cPLA2 expression and activity through calcium binding to the C2 domain, thus facilitating cPLA2 translocation to membranes, which is crucial for its function. This process is tightly modulated by phosphorylation, mainly via MAPK pathways, which phosphorylate cPLA2 and increase its enzymatic activity. Calcium/calmodulin-dependent protein kinase II (CaMKII) also phosphorylates cPLA2, further regulating its activation. Therefore, calcium influx and related kinase signalling collaboratively regulate cPLA2 expression and activity 47, 48. Furthermore, increased cPLA2 activity promotes the release of AA, thereby influencing MLC phosphorylation. For example, studies suggest that AA may indirectly regulate MLC phosphorylation via the p38-HSP27 and Rho-ROCK signalling pathways 49. However, the precise molecular mechanisms underlying this regulation require further investigation.

Regulating myosin activity through nuclear membrane-mediated mechanical transduction has unique advantages: this regulation provides a spatial organization platform for precise signal molecule localization, enables direct regulation of gene expression through nuclear membrane mechanical changes, and increases signalling specificity by avoiding interference from cytoplasmic signalling pathways. Additionally, we found that Piezo1 expression was also increased in the Nar channel (Figure 3). As a mechanosensitive ion channel, Piezo1 has been rigorously characterized through extensive studies. Its mechanosensitivity exhibits dual-layered regulation: although rapid channel activation constitutes the canonical response 50-52, Piezo1 expression levels are also dynamically modulated by mechanical stimuli 53, 54. Although Piezo1 expression regulation is not fully understood 55, we suggest two potential synergistic mechanisms: (1) Nar channel-induced cytoskeletal remodelling may upregulate Piezo1 through the activation of mechanosensitive pathways such as RhoA/ROCK 56; mechanical stimulation activates mechanosensitive ion channels (e.g., Piezo1) to mediate Ca²⁺ influx, which in turn triggers Ca²⁺/calmodulin-dependent protein kinase (CaMK) or protein kinase C (PKC) signalling pathways, subsequently activating transcription factors (e.g., NF-κB), which may upregulate Piezo1 transcription 57. (2) Nuclear mechanics: Chromatin reorganization from nuclear deformation may increase Piezo1 promoter accessibility 58, 59. These processes likely occur at different spatiotemporal scales but require higher-resolution validation. Moreover, mechanical stimulation may influence tumour cell metastasis by directly modulating actin cytoskeleton reorganization and mitochondrial dynamics, among other pathways 60, 61. Current research faces two primary limitations: existing techniques cannot resolve force‒molecular switch coupling at the nanometre scale (e.g., mechanical gating of Piezo1), necessitating the development of AFM-FRET hybrid systems; and static microchannel models inadequately represent dynamic ECM remodelling *in vivo* (e.g., ±15 kPa stiffness fluctuations in tumour stroma), highlighting the need for optogenetic hydrogel platforms with spatiotemporal control.

In conclusion, our findings reveal how nuclear membrane-mediated mechanotransduction orchestrates the interplay between nuclear mechanics and signalling pathways, providing critical insights into diseases associated with aberrant mechanical transduction. Therapeutic interventions targeting nuclear mechanical properties and associated signalling cascades may offer novel strategies for treating cell migration disorders.

## Supplementary Material

Supplementary figures.
