# Supplementary material for: Nuclear deformation acts as a mechanical switch to drive breast cancer cell migration in a confined microenvironment
Source: Theranostics. 2026 Jan 1;16(2):898–914. doi: 10.7150/thno.119211 (PMC12675006; doi:10.7150/thno.119211)
Supplement: Supplementary file 1 — Supplementary figures. [file thnov16p0898s1.pdf]

Supplementary Information

Nuclear deformation acts as the mechanical switch to drive breast cancer cell migration in confined microenvironment

Meng Wang [1], Xiaodie Li [1], Boyang Li [1], Hanyu Guo [1], Zihan Zhao [1], Xiyue Sun [1], Wei Yan [1], Yubo Tan [1], Jinyi Liu [1], Yungchang Chen [1], Shun Li [1], Tingting Li [1], Xiaozhen Dai [2*], Xiang Qin [1*], Yiyao Liu [1, 3, 4, 5*]

1. Department of Medical Oncology, Sichuan Clinical Research Center for Cancer, Sichuan Cancer Hospital & Institute, Sichuan Cancer Center, and School of Life Science and Technology, University of Electronic Science and Technology of China, Chengdu 610054, P R. China
2. Department of Otorhinolaryngology, Hospital of Chengdu University of Traditional Chinese Medicine, Chengdu 610072, P. R. China
3. Traditional Chinese Medicine (TCM) Prevention and Treatment of Metabolic and Chronic Diseases Key Laboratory of Sichuan Province, Hospital of Chengdu University of Traditional Chinese Medicine, Chengdu 610072, Sichuan, P R. China
4. Key Laboratory of Target Discovery and Protein Drug Development in Major Diseases of Sichuan Higher Education Institutes, Chengdu Medical College, Chengdu 610500, P. R. China
5. Department of Urology, Deyang People's Hospital, Deyang, Sichuan 618099, P. R. China

*Corresponding authors: Xiaozhen Dai (daixiaozhen@cmc.edu.cn); Xiang Qin (qinxiang@uestc.edu.cn); Yiyao Liu (liuyiyao@uestc.edu.cn).

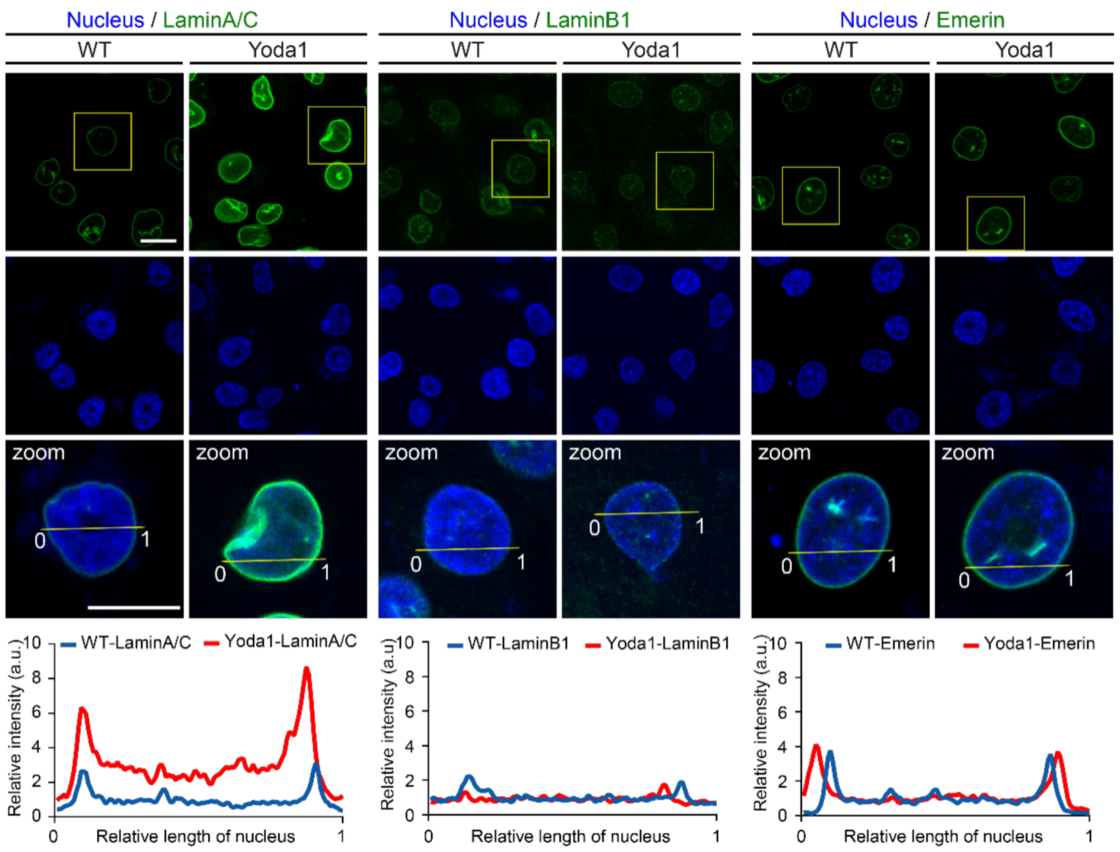

Figure S1. Fluorescence images of nuclear membrane proteins Lamin A/C, Lamin B1, and Emerin with and without Yoda1 treatment. Zoomed images of yellow boxed regions shown below. Statistical graphs show relative fluorescence intensity along yellow lines at relative nuclear lengths for each protein ($n \geq 3$). Scale bar: 20 μm.

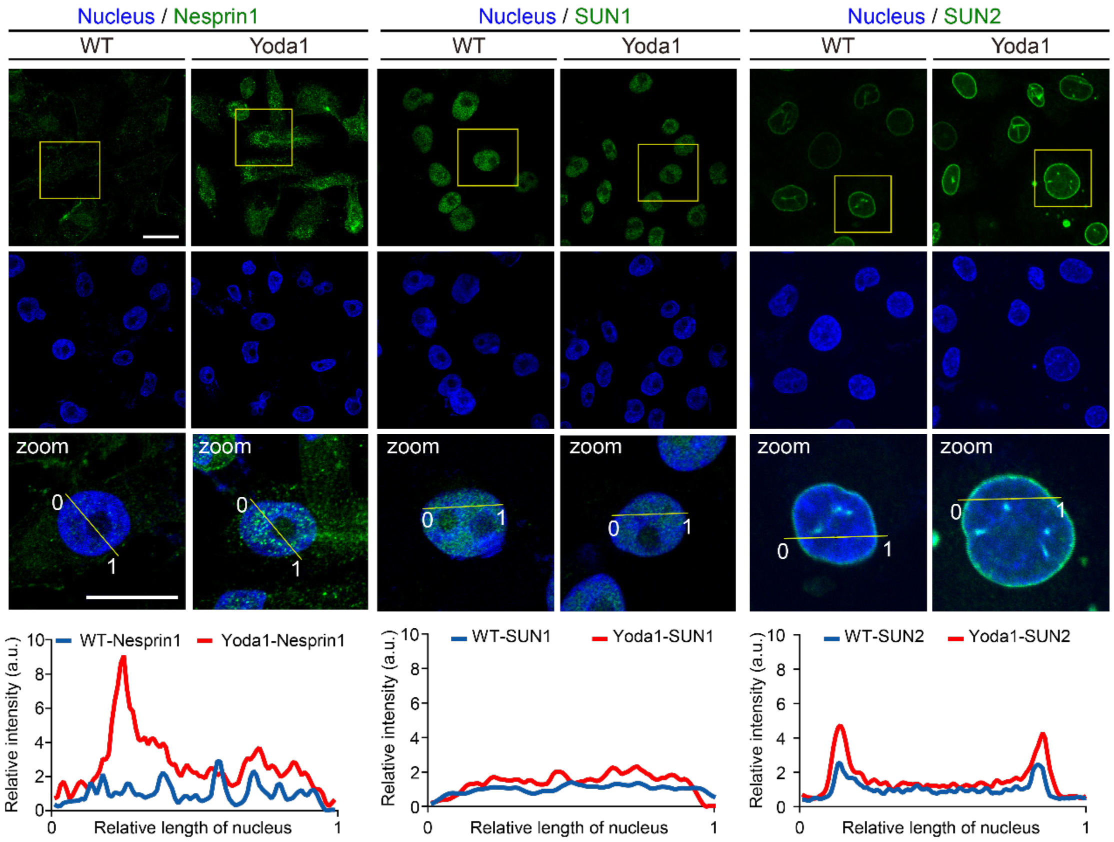

Figure S2. Fluorescence images of LINC complex components Nesprin1, SUN1, and SUN2 with and without Yoda1 treatment. Zoomed images of yellow boxed regions shown below. Statistical graphs show relative fluorescence intensity along yellow lines at relative nuclear lengths for each component ($n \geq 3$). Scale bar: 20 µm.

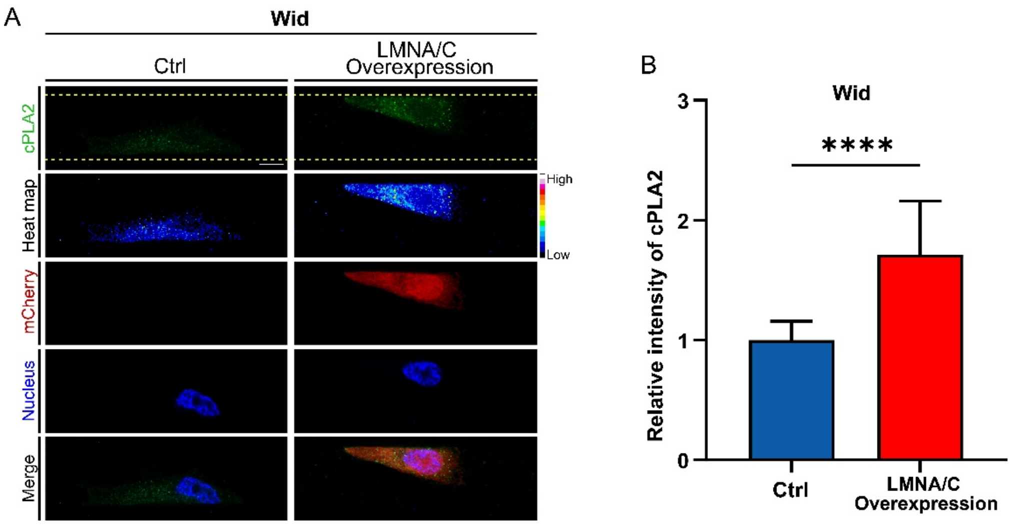

Figure S3. Effects of LMNA/C overexpression on cellular cPLA2. (**A**) Fluorescence images showing cPLA2 (green) and nucleus (blue) staining in Ctrl and LMNA/C Overexpression cells after plasmid transfection in the Wid channel, with mCherry indicating plasmid fluorescence marker. The channel edges are marked by yellow dotted lines. Scale bar: 10 μm. (**B**) Statistical analysis of relative cPLA2 fluorescence intensity in Ctrl and LMNA/C Overexpression cells ($n \geq 30$).

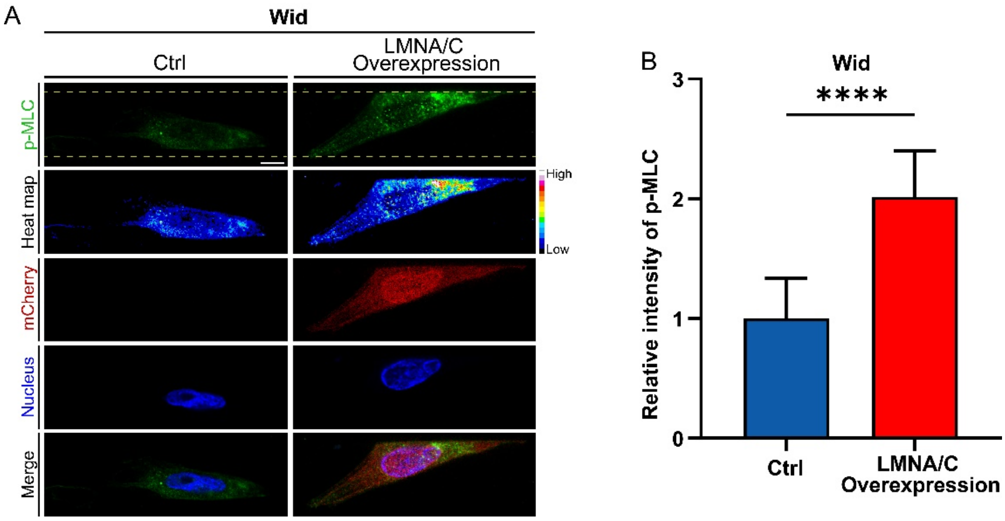

Figure S4. Effects of LMNA/C overexpression on cellular p-MLC. (**A**) Fluorescence images showing p-MLC (green) and nucleus (blue) staining in Ctrl and LMNA/C Overexpression cells after plasmid transfection in the Wid channel, with mCherry indicating plasmid fluorescence marker. The channel edges are marked by yellow dotted lines. Scale bar: 10 µm. (**B**) Statistical analysis of relative p-MLC fluorescence intensity in Ctrl and LMNA/C Overexpression cells ($n \geq 30$).

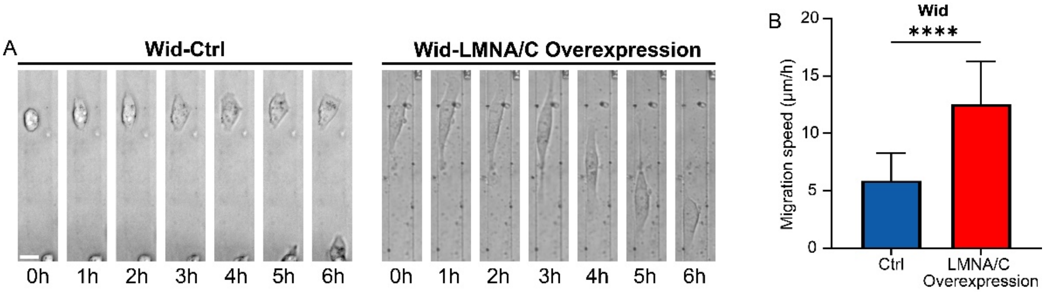

Figure S5. The cell migration situations before and after overexpression of LMNA/C in the Nar channel. (**A**) Time-lapse image segments of breast cancer cells migration in the Nar channel. Scale bar: 10 μm. (**B**) Statistics of breast cancer cells migration speed. (Statistical significance: ****$p < 0.0001$).

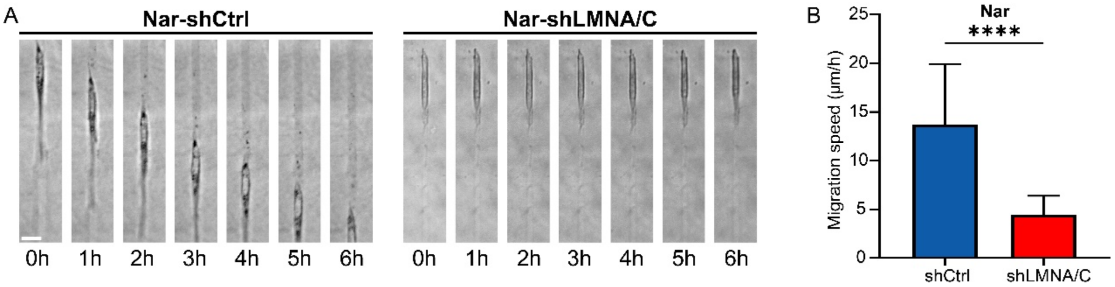

Figure S6. The cell migration situations before and after knockdown of LMNA/C in the Wid channel. (**A**) Time-lapse image segments of breast cancer cells migration in the Wid channel. Scale bar: 10 μm. (**B**) Statistics of breast cancer cells migration speed. (Statistical significance: ****$p < 0.0001$).
